# Latent profile analysis of resilience among people with serious mental illness

**DOI:** 10.1111/papt.70055

**Published:** 2026-03-15

**Authors:** Deyu Pan, Sang Qin, Wilson J. Brown, Jessica Wojtalik, Charisse Nixon, Beatrice Lee, Emre Umucu

**Affiliations:** ^1^ School of Behavioral Sciences and Education The Pennsylvania State University Middletown Pennsylvania USA; ^2^ Department of Rehabilitation Psychology and Special Education University of Wisconsin‐Madison Madison Wisconsin USA; ^3^ Department of Psychological Sciences Case Western Reserve University Cleveland Ohio USA; ^4^ Jack, Joseph, and Morton Mandel School of Applied Social Sciences Case Western Reserve University Cleveland Ohio USA; ^5^ School of Humanities and Social Sciences, The Behrend College The Pennsylvania State University Erie Pennsylvania USA; ^6^ College of Health Science The University of Texas at El Paso El Paso Texas USA

**Keywords:** adaptation, latent profile analysis, resilience, serious mental illness

## Abstract

**Objectives:**

Resilience is a critical indicator of the personal recovery process for people with serious mental illness (SMI). However, little is known about resilience subtypes among this population. Grounded in Kumpfer's resilience model (KRM), the study aims to identify latent types of resilience among people with SMI using latent profile analysis (LPA).

**Design:**

A cross‐sectional survey design was used.

**Methods:**

A total of 297 individuals with self‐reported SMI completed an online survey, including demographic variables and measures that resemble core components of the KRM.

**Results:**

The LPA identified three resilience profiles: *Maladaptive*, *Homeostatic* and *Resilient*. One‐way analyses of variance (ANOVA) revealed distinct patterns of the three resilience profiles on all factors in the KRM and the outcome variable—adaptation to psychiatric disability. ANOVA and Chi‐square tests indicated several demographic variables predict profile membership, including age, marital status, highest educational attainment, employment status, average weekly work hours and primary SMI diagnosis. However, sex, race–ethnicity, annual income and years since SMI diagnosis do not predict profile membership.

**Conclusions:**

The study contributes to the understanding of resilience subtypes and associated protective and risk factors for resilience among people with SMI, suggesting early, tailored strength‐based interventions to promote resilience and personal recovery.

## BACKGROUND

Serious mental illness (SMI) represents an umbrella term that broadly subsumes a variety of chronic, debilitating psychiatric disorders that are associated with functional disability (Gonzales et al., 2022; Ruggeri et al., 2000). The lifetime prevalence rate of SMI among U.S. adults is estimated to be 5.8% (Salzer et al., 2018). Individuals with SMI generally experience far worse outcomes on both functional and illness severity indices relative to the general population, including poorer educational and vocational attainment, community integration, and quality of life (Gibson et al., 2011; Harrison et al., 2020; Mojtabai et al., 2015; Mueser & McGurk, 2014), as well as increased morbidity, mortality, suicidality, substance use, and hospitalizations (Hayes et al., 2012; Schmutte et al., 2021; Vazquez et al., 2020; Viron & Stern, 2010), respectively. The public health burden of SMI is approximately $317 billion annually, underscoring the impetus to reduce associated functional impairments and promote personal recovery (Seabury et al., 2019).

Historically, the prognosis for individuals diagnosed with SMI is assumed to be poor, with recovery considered an unlikely outcome (Drake & Whitley, 2014; Gonzales et al., 2022; Leonhardt et al., 2017; Molstrom et al., 2022). Yet, an abundance of contemporary research challenges these limited preconceptions and indicates that a prognosis of lifelong dysfunction is often the exception, rather than the norm, among this population (see Leonhardt et al., 2017, for a review). Nearly one‐third of adults with a lifetime SMI reported the remission of their symptoms for at least the previous 12 months in a recent national study (Salzer et al., 2018). Additionally, recovery rates tend to be low until the age of 32 years, after which they begin to gradually *increase*. Such research continues to inform the operational redefinition of recovery with consideration of both subjective and objective outcomes (i.e., the personal recovery paradigm) and the search for associated prognostic indicators. One such factor that is conceptualized as the cornerstone to understanding and supporting personal recovery from SMI is resilience (Meyer & Mueser, 2011).

Resilience is traditionally defined as the ‘process, capacity, or outcome of successful adaptation despite challenges or threatening circumstances’ (Masten et al., 1990, p. 426). The concept of resilience, which comprises both the capacity to rebound in the aftermath of adversity and adapt favourably in a manner that improves functioning and quality of life (Meyer & Mueser, 2011), is particularly salient for individuals with SMI, among whom exposure to adversity and trauma is nearly ubiquitous (see Grubaugh et al., 2011, for a review). Given the chronic and pervasive nature of traumatic stress among individuals with SMI, as well as the severe functional impairments that accompany a diagnosis of a chronic psychiatric illness, identification of the specific capacity for resilience within individuals with SMI reflects a paradigm shift from deficit‐focused to strength‐based approaches and is critically warranted.

Extant research on resilience among individuals with SMI remains limited, and existing investigations of such often deem SMI samples as less resilient than ‘healthy’ groups who did not develop mental illness (Deng et al., 2018; James et al., 2013). Yet, the relatively common occurrence of personal recovery among individuals with SMI potentially implicates resilience as a critical protective factor. This is further supported by observed robust associations between resilience and less severe psychiatric symptoms, less perceived stigma, improved psychosocial functioning, and better quality of life among people with SMI (see Chuang et al., 2023, and Yeo et al., 2022, for reviews). However, studies of resilience in SMI populations included in these reviews predominantly utilize measures that conceptualize resilience as a quality rather than a multi‐faceted process. As such, the specific underlying factors that contribute to the resilience process in this population remain obscured.

### Kumpfer's resilience model

Resilience remains a challenging construct to operationalize, given its multi‐faceted and dynamic nature (Masten et al., 1990). What is agreed upon is that numerous idiographic and environmental factors interact to affect the resilience process. To account for the complex transactions between a person and their environment, Kumpfer (2002) proposed the person–process–context resilience model, which includes four domains of influence (i.e., acute stressor or challenge, environmental context, individual characteristics, outcome), as well as two transactional junctures between two domains (i.e., person–environment interaction, resilience process).

According to Kumpfer, resilient individuals actively shape their environment through the transactional points in the model over time, which increasingly lead to positive outcomes. Each introduction of a new acute stressor disrupts the present homeostasis and necessitates the activation of the resilience process. Depending on how successfully individuals adapt to these stressors, Kumpfer (2002) proposed three distinct resilience outcomes: maladaptive reintegration, homeostatic reintegration, and resilient reintegration. Maladaptive reintegration reflects unsuccessful adaptation and diminished functioning, homeostatic reintegration represents a return to baseline functioning without substantial growth, and resilient reintegration involves positive adaptation and strengthened functioning following adversity.

The achievement of positive outcomes depends on whether adaptation occurs amid the convergence of environmental context, individual characteristics, and person–environment interactions (Kumpfer, 2002). As applied to individuals with disability (e.g., SMI), maladaptation presumably results when person–environment interactions are compromised or ineffective (Wright, 1983), whereas resilience characterizes the process by which the individual competently manages adversity through such person–environment interactions (Egeland et al., 1993).

While some recent studies offer support for the constructs of Kumpfer's Resilience Model (KRM) in the prediction of adaptation across disability samples (i.e., people with spinal cord injury, inflammatory bowel disease; Luo et al., 2019; Tansey et al., 2017), few do so in individuals with SMI. To date, there is only one known study of KRM applied to SMI (i.e., Pan & Sánchez, 2022): They examined the contribution of every core component of KRM to the adaptation to psychiatric disability (i.e., outcome) using hierarchical regression. The final hierarchical regression model explained 71% of the variance in the adaptation to disability, offering strong support for the validity of KRM among individuals with SMI (Pan & Sánchez, 2022). Importantly, all but one component were significant predictors in the final model, while person–environment interaction was the strongest predictor, consistent with Kumpfer's emphasis on this transactional dynamic as a critical determinant for resilience (Kumpfer, 2002). Identifying resilience subtypes based on the KRM, a comprehensive theoretical framework with operationalized and validated components, might be particularly beneficial to inform early, individualized intervention efforts that bolster resilience and promote adaptive response to adversity among individuals with SMI.

### KRM and resilience subtypes

Multiple investigations establish precedents for the examination of resilience subtypes from the lens of KRM. For example, using latent profile analysis (LPA), four types of resilience were recently identified in gastric cancer survivors: low, moderate, medium‐high, and high (Wang et al., 2024). Another study identified low, moderate, and high resilience profiles among lung cancer survivors (Li et al., 2024). However, the variables included in both studies were not comprehensively representative of KRM. Numerous other studies use LPA to examine resilience subtypes in various populations (e.g., trauma‐exposed children and adolescents, healthcare workers, university students, Veterans, etc.), but without an organizing theoretical framework (see Janousch et al., 2022; Klingenberg & Süß, 2022; Nordstrand et al., 2024; Wang et al., 2021). Despite high prevalence rates of trauma exposure, psychiatric symptom severity, and functional impairments, to date, no study has comprehensively examined all KRM components to identify resilience subtypes among individuals with SMI.

### Current study

Resilience is a critical prognostic indicator of personal recovery in persons diagnosed with SMI, yet the investigation of such in this population remains limited. The identification of resilient subtypes in individuals with SMI holds the potential to support early, tailored strength‐based interventions to promote resilience and personal recovery (Meyer & Mueser, 2011). Yet, research investigations of such require careful, theory‐driven observations of related variables to reveal unique subgroups of resilience. KRM was selected as the guiding framework because its components have been empirically validated in SMI populations in a manner consistent with Spurk et al.'s (2020) recommendations that profile indicators be theoretically related yet distinct. Prior study (i.e., Pan & Sánchez, 2022) has demonstrated that KRM components uniquely contribute to outcomes while interconnecting coherently, supporting their suitability for LPA.

KRM also offers a comprehensive understanding of the core factors and transactional dynamics that contribute to the process of resilience. To the authors' knowledge, this study will be the first to comprehensively examine variables operationalizing each component of KRM together to identify latent types of resilience among people with SMI. Current study aims include the following research questions: (a) What is the nature of each latent profile group identified? (b) What demographic variables predict profile membership? And (c) Are there differences in adaptation to psychiatric disability among identified profiles?

## MATERIALS AND METHODS

### Procedures

Data used in this study were obtained from a larger cross‐sectional study that examined the resilience process among people with SMI (Pan & Sánchez, 2022). The original study was reviewed and approved by the Institutional Review Board at The University of Iowa (Approval ID: 201908749). Additional ethical review and approval were waived for secondary analysis on a deidentified dataset in accordance with the first author's institutional policy. Participant recruitment was conducted on ResearchMatch.org, a National Institute of Health‐funded volunteer registry that connects volunteers with various health conditions with clinical research studies (Harris et al., 2012). Recruitment Access on ResearchMatch requires IRB approval and an IRB‐approved contact message, with recruitment activities overseen by institutional liaisons. Invitations are routed through a secure clearinghouse, and volunteer contact information is released only after participants indicate interest and authorize release.

Working‐age adults (between 18 and 65 years old) who indicated having an SMI diagnosis in their Research Match profile were invited to participate. Prescreening was conducted to identify qualified potential participants, and emails were sent to identified individuals with a Qualtrics survey link. Participants who consented to participate would proceed to complete demographic questions and several validated self‐report measurements. At the end of the survey, participants can choose to enter a drawing to receive 1 of 200 possible $10 Amazon gift cards as compensation for their participation. To improve data quality and reduce fraudulent responses, unique single‐use survey links, duplicate detection, and bot detection features of Qualtrics were enabled, and attention‐check items were embedded in the survey. Responses that failed attention checks or showed evidence of fraudulence were flagged and excluded from data analysis. These procedures align with published recommendations and prior reports describing the detection of fraudulent responses in ResearchMatch‐recruited web‐based studies (Pageau & Ling, 2025; Pozzar et al., 2020).

### Participants

All participants were asked to select all applicable psychiatric diagnoses from a predefined list. When multiple diagnoses were endorsed, the survey prompted participants to identify a single primary diagnosis; however, no standardized definition or criteria were provided for determining which diagnosis should be considered primary. To determine study eligibility, participants were then asked if the mental illness substantially interferes with or limits one or more major life activities (e.g., self‐care, interpersonal relationships, employment, schooling). This screening item aligned with the definition of SMI by the National Institute of Mental Health (NIMH, 2024). Participants who responded ‘no’ to this question were excluded from the study. Of the 428 participants who initiated the survey in spring 2021, 131 were excluded due to incompletion or having a mental illness, but not SMI. The final sample was composed of 297 individuals with a mean age of 31.49 (*SD* = 12.66). Specifically, most self‐reported being diagnosed by a mental health professional with major depressive disorder (*n* = 78), followed by anxiety disorders (*n* = 60), bipolar disorders (*n* = 55), post‐traumatic stress disorder (PTSD; *n* = 55), and others (e.g., schizophrenia spectrum disorders, obsessive‐compulsive disorder, personality disorders; *n* = 19). The majority of participants (80.7%) reported more than one psychiatric diagnosis. The average number of years since being diagnosed with SMI was 10.73 (*SD* = 9.75). Participants also reported working 25.36 h per week on average, but with large variations (*SD* = 18.91). For other demographic characteristics of the sample, please refer to Table 1.

**TABLE 1:** Participant demographic characteristics (*N* = 297).

Demographic variables	*n*/*M*	%/*SD*
Sex		
Male	41	13.80
Female	247	83.16
Race/ethnicity		
White	225	75.76
Black	6	2.02
Asian	14	4.71
Hispanic	16	5.39
Other/Biracial	11	3.70
Education level		
High school degree/Some college but no degree	107	36.03
Associate/Bachelor's degree	120	40.40
Graduate degree	61	20.54
Employment status		
Employed (full‐time or part‐time)	133	44.78
Unemployed	31	10.44
Student	111	37.37
Others	13	4.38
Average weekly work hour	25.36	18.91
Annual income		
Less than $10,000	88	29.63
$10,000–$19,999	45	15.15
$20,000–$29,999	30	10.10
$30,000–$39,999	37	12.46
$40,000–$49,999	28	9.43
$50,000–$59,999	27	9.09
$60,000 or above	31	10.44
Marital status		
Married (domestic partnership)	61	20.54
Widowed/divorced/separated	38	12.79
Never married	189	63.64
Primary diagnosis		
Bipolar	55	18.52
Depression	78	26.26
Anxiety	60	20.20
Post‐traumatic stress disorder	55	18.52
Personality disorder	30	10.10
Others	19	6.40
Years since being diagnosed with SMI	10.73	9.75

### Measures

The demographic variables examined in this study were sex, marital status, race and ethnicity, highest educational attainment, employment status, annual income, average weekly work hours and years since diagnosed with SMI. The independent variables (i.e., profile indicators of resilience) and outcome variable (i.e., adaptation to psychiatric disability) investigated in this study collectively resemble the six major components (i.e., four domains and two transactional processes) of the KRM (Kumpfer, 2002):

1. Stressors (i.e., psychiatric symptom severity);
2. Environmental Context (i.e., perceived public stigma);
3. Person–Environmental Transactional Process (i.e., self‐stigma);
4. Internal Resiliency Factors (i.e., hope, optimism, self‐efficacy, and neuroticism personality trait);
5. Resiliency Process (i.e., resilience);
6. Positive Life Outcomes (i.e., adaptation to psychiatric disability).

#### Profile indicators

##### Stressors

The *Symptom Checklist‐9* (SCL‐K‐9; Klaghofer & Brähler, 2001), a 9‐item self‐report measure of psychiatric symptom severity, was used to represent the Stressors component of the KRM. Participants indicated how much they were bothered by specific psychiatric symptoms (e.g., ‘feeling nervous when you are left alone’) in the past week on a 5‐point Likert scale from 0 (*Not at all*) to 4 (*Extremely*). Higher total scores correspond with higher levels of psychiatric symptom severity. The SCL‐K‐9 exhibits promising psychometric properties among people with SMI (Pan, Qin, et al., 2023). In the present study's sample, the coefficient alpha was .84.

##### Environmental context

The *Perceived Discrimination and Devaluation scale* (PDD‐8; Link et al., 2008), an 8‐item measure of perceived public stigma, was used to reflect the Environmental Context component of the KRM. Participants rated their agreement with each item (e.g., ‘Most people think less of a person who has received mental health treatment’) on a 6‐point Likert scale (1 = *Strongly disagree* to 6 = *Strongly agree*). The higher the total scores, the greater the level of perceived public stigma. The PDD‐8 demonstrates good validity and reliability (Link et al., 2008). In the present study's sample, the coefficient alpha for the PDD‐8 was .83.

##### Person–environment interactional processes

The *Self‐Stigma Scale‐Short* (SSS‐S; Mak & Cheung, 2010), a 9‐item measure of self‐stigma, was used to correspond to the Person–Environment Interactional Processes component of the KRM. Participants rated items (e.g., ‘I fear that others would know that I am a mental health consumer’) on a 4‐point Likert‐type scale from 1 (*strongly disagree*) to 4 (*strongly agree*). The higher the average scores, the greater the self‐stigma. The SSS‐S demonstrates satisfactory psychometric properties among people with SMI (Pan, Babb, et al., 2023). In the present study's sample, the coefficient alpha for the SSS‐S was .89.

##### Internal self‐characteristics

Hope, optimism, self‐efficacy, and the neuroticism personality trait collectively represent the Internal Self‐Characteristics component of the KRM.

The *Adult Trait Hope Scale* (ATHS; Snyder et al., 1991) is an 8‐item scale that assesses hope. Participants rated each item (e.g., ‘I energetically pursue my goals’) on an 8‐point Likert‐type scale from 1 (*Definitely False*) to 8 (*Definitely True*). Higher scores represent greater hope. The ATHS demonstrates satisfactory psychometric properties among various samples (Snyder et al., 1991). In the present study's sample, the coefficient alpha for the ATHS was .88.

The *Life Orientation Test‐Revised* (LOT‐R; Scheier et al., 1994) is a 6‐item scale that measures optimism. Participants rated each item (e.g., ‘In uncertain times, I usually expect the best’) on a 5‐point Likert‐type scale from 0 (*strongly disagree*) to 4 (*strongly agree*). The higher the total scores, the greater the optimism. The LOT‐R exhibits acceptable psychometric properties (Scheier et al., 1994). Coefficient alpha for the LOT‐R in the current sample was .87.

A 6‐item version of the *Generalized Self‐Efficacy Scale* (GSE‐6; Romppel et al., 2013) is used to measure general self‐efficacy. Participants rated each item (e.g., ‘No matter what comes my way, I am usually able to handle it.’) on a 4‐point Likert‐type scale from 1 (*not at all true*) to 4 (*exactly true*). The higher the total scores, the greater the self‐efficacy. The GSE‐6 possesses acceptable psychometric properties (Romppel et al., 2013). In the present study's sample, the coefficient alpha for the GSE‐6 was .83.

The *Neuroticism* subscale of the *Big Five Inventory* (BFI‐N; John & Srivastava, 1999) is an 8‐item measure of the Neuroticism personality trait. Participants rated each item (e.g., ‘I see myself as someone who worries a lot’) on a 5‐point Likert‐type scale from 1 (*Disagree Strongly*) to 5 (*Agree Strongly*). Higher scores reflect higher levels of neuroticism. The BFI‐N generally demonstrates good psychometric properties (John et al., 2008; John & Srivastava, 1999). In the present study's sample, the coefficient alpha for the BFI‐N was .81.

##### Resilience processes

The *Brief Resilience Scale* (BRS; Smith et al., 2008), a 6‐item self‐report instrument of resilience, was used to operationalize the Resilience Process of the KRM. Participants rated each item (e.g., ‘I usually come through difficult times with little trouble’) on a 5‐point Likert‐type scale from 1 (*strongly disagree*) to 5 (*strongly agree*). Negative‐worded items were reverse‐scored so that higher scores indicate greater resilience. The BRS exhibits satisfactory psychometric properties among different samples (Sánchez et al., 2021; Smith et al., 2008). In the present study's sample, the coefficient alpha was .89.

### Outcomes variable

The *Adaptation to Disability Scale‐Revised‐23* (ADS‐R‐23; Sánchez et al., 2016), a 23‐item measure of adaptation to psychiatric disabilities, assessed the outcome component of the KRM. Participants rated each item (e.g., ‘Mental health problems or not, I am going to make good in life.’) on a 4‐point Likert‐type scale from 1 (*strongly disagree*) to 4 (*strongly agree*), with higher scores corresponding to greater adaptation to psychiatric disability. The ADS‐R‐23 demonstrates excellent psychometric properties among adults with SMI (Sánchez et al., 2016). Coefficient alpha for the ADS‐R‐23 in the current sample was .94.

### Data analysis

First, an LPA was conducted to generate 1‐ to 5‐profile models based on eight measures of the profile indicators on M*plus* (version 8.4). LPA is a data analytic approach that identifies latent subgroups within a larger population based on specific observed variables (Spurk et al., 2020). LPA is particularly well‐suited for the detection of unique patterns across constructs with multiple facets, such as resilience. As applied to observable data that represent each of the six components of KRM, LPA may produce theory‐driven resilience profiles that reflect distinct resilience characteristics. LPA also permits the analysis of Kumpfer's factors holistically, consistent with the transactional nature of KRM, wherein factors continuously converge and interact to influence outcomes (Kumpfer, 2002). Consistent with the person‐centred and transdiagnostic nature of LPA, participants' psychiatric diagnoses were not used as a profile indicator to generate the latent profiles. Given the high prevalence of psychiatric comorbidity in the sample and the study's focus on the resilience process rather than diagnostic classification, including diagnostic categories in profile generation could have obscured meaningful cross‐diagnostic patterns. Psychiatric diagnosis was therefore treated as descriptive and examined outside of the LPA generation process. The maximum likelihood with robust standard errors (MLR) estimator was utilized due to non‐normal multivariate distributions of the profile indicators; full information maximum likelihood (FIML) estimation was used to handle missing data (Spurk et al., 2020). As a priori power analysis for LPA is complicated, sample size adequacy was evaluated based on model convergence, stability, and interpretability, consistent with existing methodological guidance (Nylund et al., 2007; Spurk et al., 2020).

Next, a model comparison approach was taken to determine the best‐fitting profile model based on theoretical considerations and several statistical fit indices. Specifically, lower values on Bayesian information criterion (BIC), sample‐size adjusted BIC (SABIC) and Akaike information criterion (AIC) are suggestive of a better model fit; significant results on Bootstrap likelihood ratio test (BLRT) and the adjusted Lo–Mendell–Rubin (adjusted LMR) tests suggest that the estimated model fits the data better than the model with one less latent profile and higher entropy values indicate more accurate classification of models (Ram & Grimm, 2009). The sample size of each profile was reviewed within the estimated models, which were rejected with a profile with less than 1% of the total sample size or 25 cases (Spurk et al., 2020).

After retaining the optimal profile solution, each participant was classified into one of the profiles based on their distinctive patterns of the KRM components. To better understand the profile memberships, one‐way analyses of variance (ANOVAs) with Bonferroni corrections were conducted on all profile indicators and the outcome variable across different latent profile groups with IBM SPSS Statistics Version 29. A series of ANOVAs for continuous variables (i.e., age, annual income, average weekly work hours, years since diagnosed with SMI) and chi‐square (*χ* ^2^) tests for categorical variables (i.e., sex, marital status, race and ethnicity, highest educational attainment, employment status, and primary SMI diagnosis) were also conducted to compare profile differences in demographic variables.

## RESULTS

### LPA

The fit indices for the 1‐ to 5‐profile models are provided in Table 2. The SABIC and the AIC continued to decrease as the specified profile group number increased, suggesting better fits for more complex models. The BIC continued to decrease until the 4‐profile model and observed an increase for the 5‐profile model, suggesting that the 4‐profile model was the best fit for the data. However, entropy was the highest for the 3‐profile model. The BLRTs were significant for all models. The Adjusted LMR tests were no longer significant for the 4‐ and 5‐profile models, indicating the 3‐profile model fits better than the 4‐profile model. Furthermore, a class with fewer than 25 cases was observed for the 5‐profile model. Considering these fit statistics, the 3‐profile model was chosen as the final fitted model.

**TABLE 2:** Model fit statistics for LPA with different latent profiles.

Models	AIC	BIC	SABIC	Adjusted LMR (*p*)	BLRT (*p*)	Entropy
1	5607.24	5666.34	5615.60	–	–	–
2	5079.32	5171.66	5092.38	0.00	<0.0001	0.80
3	4864.81	4990.39	4882.57	0.00	<0.0001	0.85
4	4810.43	4969.26	4832.89	0.08	<0.0001	0.79
5	4787.57	4979.64	4814.73	0.54	<0.0001	0.78

Abbreviations: Adjusted LMR, adjusted Lo–Mendell–Rubin; AIC, Akaike information criterion; BIC, Bayesian information criterion; BLRT, Bootstrap likelihood ratio test; SABIC, sample‐size adjusted BIC.

Each participant was categorized into 1 of 3 mutually exclusive groups based on the highest posterior probabilities generated for each of the three profile groups: 34 participants (11.45% of the total sample) were grouped in *Profile 1*, 171 (57.58%) in *Profile 2*, and 92 (30.98%) in *Profile 3*. The average posterior probability for those assigned to the corresponding profile was 95.2%, 91.9%, and 94.0% for *Profile 1*, *Profile 2*, and *Profile 3*, respectively, suggesting that the results of the three‐latent‐profile model were reasonable (Sinha et al., 2021).

**FIGURE 1:**
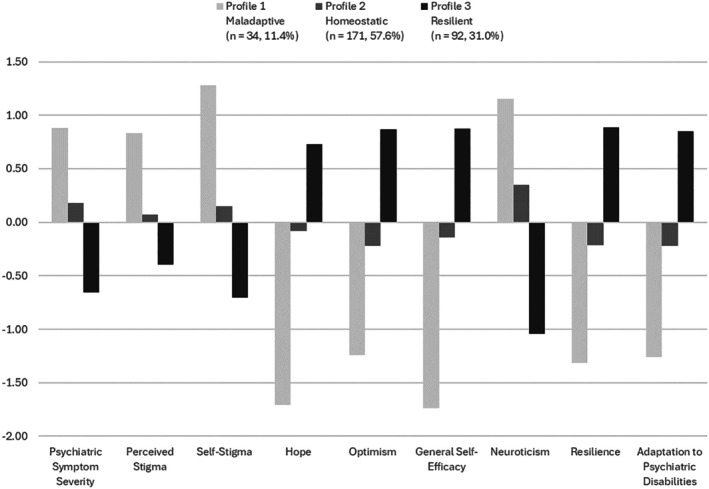
*Z*‐scores of all profile indicators and outcome variables for each profile in the 3‐profile model.

Figure 1 provides a visual representation of standardized scores on all profile indicators and the outcome variable for the three profiles in the retained 3‐profile model. Based on the observed patterns among the three profile groups, each profile was named to represent the three distinct resilience types proposed by Kumpfer (2002): Profile 1 was named *Maladaptive*, Profile 2 was named *Homeostatic* and Profile 3 was named *Resilient*. Profile 1 (Maladaptive) was characterized by the highest scores on psychiatric symptom severity, perceived public stigma, self‐stigma and neuroticism and the lowest scores on hope, optimism, general self‐efficacy and resilience, while Profile 3 (Resilient) was characterized by the lowest scores on psychiatric symptom severity, perceived public stigma, self‐stigma and neuroticism, and the highest scores on hope, optimism, general self‐efficacy and resilience. Profile 2 (Homeostatic) was generally more moderate than the extremes of the other two profiles, exhibiting similar patterns but without reaching their level of extremity.

### Profile differences in resilience characteristics

Table 3 summarizes the results of ANOVAs with profile membership as the independent variable. All the *F* values were statistically significant (*p* < .05). Specifically, post‐hoc Bonferroni comparisons showed that the differences among the three profiles on all profile indicators and the outcome variable were all statistically significant.

**TABLE 3:** Analysis of variance (ANOVA) results with profile membership as independent variable (*N* = 297).

KRM component	Variable	Measure	Total		Profile						*F*	*p*	Post‐hoc comparison
					Profile 1 maladaptive		Profile 2 homeostatic		Profile 3 resilient				
			*M*	*SD*	*M*	*SD*	*M*	*SD*	*M*	*SD*			
Stressors	Psychiatric Symptom Severity	SCL‐K‐9	1.81	0.84	2.55	0.73	1.96	0.73	1.26	0.74	47.12	<.001	1 > 2 > 3*
Environmental context	Perceived Stigma	PDD‐8	3.99	0.89	4.73	0.93	4.05	0.79	3.64	0.87	21.33	<.001	1 > 2 > 3*
Person–environment interactional processes	Self‐Stigma	SSS‐S	2.44	0.68	3.31	0.50	2.54	0.58	1.96	0.54	71.36	<.001	1 > 2 > 3*
Internal self‐characteristics	Hope	ATHS	5.36	1.33	3.09	1.07	5.25	0.99	6.33	0.83	132.26	<.001	1 < 2 < 3*
	Optimism	LOT‐R	1.72	0.92	0.58	0.54	1.52	0.72	2.52	0.68	113.23	<.001	1 < 2 < 3*
	General Self‐Efficacy	GSE‐6	2.66	0.57	1.67	0.41	2.58	0.35	3.16	0.33	220.52	<.001	1 < 2 < 3*
	Neuroticism	BFI‐N	3.79	0.72	4.62	0.34	4.04	0.44	3.04	0.56	187.35	<.001	1 > 2 > 3*
Resiliency processes	Resilience	BRS	2.53	0.89	1.36	0.43	2.34	0.69	3.32	0.61	127.81	<.001	1 < 2 < 3*
Outcome variable	Adaptation to Psychiatric Disabilities	ADS‐R‐23	2.77	0.54	2.09	0.40	2.65	0.41	3.23	0.42	107.51	<.001	1 < 2 < 3*

Abbreviations: ADS‐R‐23, Adaptation to Disability Scale‐Revised‐23; ATHS, Adult Trait Hope Scale; BFI‐N, Big Five Inventory – Neuroticism; BRS, Brief Resilience Scale; GSE‐6, Generalized Self‐Efficacy Scale‐6; LOT‐R, Life Orientation Test‐Revised; PDD‐8, Perceived Discrimination and Devaluation Scale‐8; SCL‐K‐9, Symptom Checklist‐9; SSS‐S, Self‐Stigma Scale‐Short.

* *p* < .05.

### Profile differences in demographic variables

Chi‐square tests indicated significant associations between profile membership and marital status (*χ* ^2^(4) = 12.08, *p* < .05), highest educational attainment (*χ* ^2^(4) = 16.06, *p* < .01), employment status (*χ* ^2^(6) = 21.32, *p* < .01), and primary SMI diagnosis (*χ* ^2^(10) = 20.76, *p* < .05). Specifically, individuals with a Maladaptive profile were more likely to have a high school diploma/some college experience but no degree, be unemployed, and exhibit a primary diagnosis of an anxiety disorder or personality disorder but less likely to have graduate degrees and primary diagnoses of depression or PTSD. Individuals with a Homeostatic profile were most likely to be never married and a current student, but less likely to have an associate or bachelor's degree, be employed, and exhibit a primary diagnosis of bipolar disorder. Individuals with a Resilient profile were more likely to be married or widowed/divorced/separated, have an associate, bachelor's, or graduate degree, be employed full‐time or part‐time, and have a primary diagnosis of depression or PTSD, but less likely to be never married and exhibit a primary diagnosis of an anxiety disorder or personality disorder. However, no significant association was found between profile membership and sex (*χ* ^2^(4) = 6.10, *p* = .19) and race–ethnicity (*χ* ^2^(8) = 4.87, *p* = .77).

ANOVA with age as the dependent variable was statistically significant (*F*_2,281_ = 6.65, *p* < .05). Post‐hoc Bonferroni tests revealed that individuals in the Resilient (*M*_age_ = 35.38) reported significantly higher age than those in the Homeostatic (*M*_age_ = 29.39) but not the Maladaptive (*M*_age_ = 32.03) profile. ANOVA with average weekly work hours as the dependent variable was statistically significant (*F*_2,258_ = 4.03, *p* < .05). Post‐hoc Bonferroni tests revealed that individuals in the Resilient (*M*_wh_ = 29.42) reported significantly more work hours per week than those in the Maladaptive (*M*_wh_ = 18.59) but not Homeostatic (*M*_wh_ = 24.28) profile. ANOVAs with annual income and years since SMI diagnosis as the outcomes did not result in statistically significant differences across profiles (*F*_2,283_ = 2.47, *p* = .087; *F*_2,229_ = 2.62, *p* = .075, respectively).

## DISCUSSION

The current study sought to examine latent profiles of resilience among individuals with SMI that align with Kumpfer's theoretical model. Understanding the resilience framework in this vulnerable population can enhance services and intervention programs, supporting individuals towards desired psychosocial adaptation outcomes. KRM encompasses four domains (i.e., stressors, environmental context, internal resiliency factors, and outcome) and two transactional processes (i.e., person–environment processes, resiliency processes) that contribute to resilience, a critical prognostic indicator of personal recovery for SMI. As a crucial transition from theory to empirical investigation, each component was operationalized in the current study to investigate potential resilience profiles in individuals with SMI, as well as whether such profiles are theoretically consistent with KRM.

Three distinct subgroups identified in this study, characterized by unique resilience patterns, mapped closely onto the three resilience outcomes proposed by Kumpfer (2002). Specifically, Profile 1, termed the ‘Maladaptive’ group, included 34 individuals (11.45% of the sample) exhibiting the most severe psychiatric symptoms, which were closely associated with heightened perceived public stigma, compatible with existing research (Gaebel et al., 2006). Frequently receiving devaluation from one's environment can lead individuals to internalize these negative views (Corrigan et al., 2011). This internalization (i.e., self‐stigma) was linked to diminished hope and optimism, a decreased belief in the capacity to achieve personal goals (i.e., self‐efficacy), and elevated levels of neuroticism. Consequently, these individuals tended to show lower resilience in responding to the challenges, which can result in adverse effects on psychosocial adaptation (Qin et al., 2023).

In contrast, Profile 3, termed the ‘Resilient’ group, was comprised of 92 individuals (30.98% of the sample) who experienced mild symptoms and perceived lower levels of public stigma. These factors contributed to lower levels of self‐stigma. Additionally, these individuals reported the highest levels of hope, optimism, and self‐efficacy among the three groups. Neuroticism traits were less prevalent in this subset as well. In conjunction with the least challenging social environment and the strongest protective internal factors, these individuals were more likely to report resilience in the face of adversity and effective adaptation to their conditions. Between Profiles 1 (Maladaptive) and 3 (Resilient) lies an intermediate group (i.e., Profile 2), coined as the ‘Homeostatic’ group to reflect Kumpfer's (2002) theoretical construct of homeostatic reintegration (i.e., a pattern of adaptation characterized by maintenance of baseline functioning without substantial growth following adversity), which consists of 171 individuals (57.58% of the sample). Consistent with the model, people in this group reported moderate levels of psychiatric symptoms, perceived public stigma, and self‐stigma. They also demonstrated median levels of hope, optimism, and self‐efficacy. They exhibited some neuroticism, though not to extreme levels. In the absence of significant stressors, they managed to balance adversity with their available internal protective resources. Thus, they tended to endorse somewhat lower levels of resilience than Profile 3 (Resilient) individuals but faced relatively minor difficulties in achieving psychosocial adaptation and psychological growth. At the same time, the ordering of profiles suggests a continuum of resilience ranging from maladaptive to resilient functioning, with the Homeostatic profile occupying an intermediate position.

Importantly, levels of resilience across the three profiles appeared to be inversely related to symptom severity, such that Profile 3 (Resilient) was associated with the lowest levels of psychiatric symptoms, whereas Profile 1 (Maladaptive) was associated with the highest levels. Because the current study is cross‐sectional, inferences about temporal associations between resilience and symptom severity are limited; however, existing research suggests a complex, reciprocal relationship between these related yet conceptually distinct constructs (Luthar & Cicchetti, 2000; Wu et al., 2013). A recent systematic review of 37 studies found that the highest levels of resilience are often observed in individuals with less severe psychiatric symptoms (Yeo et al., 2022), consistent with our results. Additionally, intact insight and fewer cognitive deficits have also been linked to higher resilience among individuals with SMI (Mizuno et al., 2016). Some evidence also suggests that resilience may mediate pathways from internal processes and clinical variables to SMI onset, functional impairment, and quality of life (Yeo et al., 2022). KRM provides a framework for understanding this relationship, which unfolds through transactional processes across time. For example, resilience processes can buffer symptom impact and promote recovery (e.g., improved adherence, reappraisal, engagement with supports), while high symptom burden can erode assets and resources (e.g., reduced self‐efficacy, strained supports), thereby weakening resilience. These processes can operate concurrently and shift over time. Thus, the apparent inverse association at the group level likely reflects an average over heterogeneous within‐person trajectories that will continue to evolve. To investigate this dynamic more precisely, future studies should model within‐person change longitudinally within samples stratified or matched on symptom burden.

Overall, the emergence of these three profiles (Maladaptive, Homeostatic, Resilient) provides empirical support for KRM's core typology within an SMI population and suggests that KRM's theorized resilience patterns can be meaningfully identified using person‐centred analytic approaches. In the meantime, the profiles varied primarily in degree rather than kind, which may indicate that resilience processes in SMI exist along a spectrum of adaptation. The collective emergence of these profiles emphasizes that resilience is not merely an external or internal process, but a dynamic interaction of both, echoing the points made by Wright (1983) and Kumpfer (2002). To achieve positive resilience outcomes for Maladaptive and Homeostatic groups, as seen in Profile 3 (Resilient), multiple intervention points may be available. For instance, mitigating contact with social environments where stigma is prevalent and fostering healthy interactions between individuals and their surroundings can be effective strategies: Anti‐stigma programs aimed at reducing public misconceptions (Corrigan et al., 2012) and preventing the development of self‐stigma (Yanos et al., 2015) were found to be effective in reducing public stigma and self‐stigma (Waqas et al., 2020). Furthermore, connecting individuals with peer support specialists may strengthen internal capacity to buffer external stressors. Peer support specialists were found to provide ‘recovery role models’ that instill hope and optimism about recovery and promote recovery self‐efficacy (Davidson et al., 2012). Additionally, while personality tendencies like neuroticism may be less amenable to intervention, they hold predictive power in identifying individuals who may struggle more with their symptoms and stigma. These findings suggest that more resources should be directed towards these individuals in the Maladaptive profile to increase their opportunities for resilience.

The current study further explored the associations between profile membership and important demographic characteristics. Specifically, individuals in the Homeostatic and Resilient groups were more likely to achieve higher educational attainment and better employment prospects. Research on resilience highlights its role as a protective factor, enabling individuals to effectively manage demands in the workplace (Shatté et al., 2017) and academic settings (Lytle & Shin, 2023). With relatively higher employment rates, individuals in the more resilient profiles worked more hours per week, indicating increased social contact and potential opportunities for meaningful social participation (Khalema & Shankar, 2014). Gainful employment also offers supportive and beneficial environmental factors that facilitate resilience (Thomas et al., 2020) and recovery (Wallstroem et al., 2021).

Moreover, our findings indicated significant age differences across resilience profiles. Participants in the Resilient profile were significantly older than those in the Homeostatic profile, but not significantly older than those in the Maladaptive profile. Individuals in the Resilient group were more likely to be married (in a domestic partnership), widowed, divorced, or separated, suggesting a higher likelihood of sustaining long‐term romantic relationships. This pattern may partially reflect age or cohort effects, particularly relative to the Homeostatic group. At the same time, age alone does not fully explain resilience differences, given the lack of a significant age difference between the Resilient and Maladaptive profiles. With obvious benefits for resilience, the ability to maintain long‐term relationships may also reflect a higher level of functionality necessary for achieving resilience (Pan & Sánchez, 2022). However, because relationship status was assessed using categorical labels and did not capture relationship duration or quality, these findings should be interpreted cautiously.

Further, although diagnostic differences across profiles were observed, diagnoses typically associated with lower perceived public stigma did not appear to determine profile membership. For example, a primary diagnosis of depression was more prominent in Profile 3 (Resilient), whereas anxiety disorders were more common in Profile 1 (Maladaptive). This finding suggests that the diagnostic label alone is insufficient to explain resilience differences. Rather, the higher prevalence of anxiety disorders in the Maladaptive profile may reflect chronic or secondary anxiety symptoms associated with poor adaptation to SMI and cumulative adversity, rather than diagnosis‐specific effects per se.

### Limitations and future research

Several limitations should be considered when interpreting the findings of this study. First, the cross‐sectional nature of the dataset limits the inference of temporal and causal relationships between variables. Although the KRM conceptually suggests sequential occurrence of components (e.g., that external pressures and internal resources precede psychosocial adaptation), the design of our study does not empirically validate this temporal sequence, nor does it exclude unmeasured confounding variables that may covary and explain relationships among these components. For example, factors such as trauma exposure, treatment engagement, cognitive functioning, and socioeconomic status were not assessed and may influence resilience processes. Future research should incorporate these variables, consider collecting longitudinal data, and employ latent trajectory analysis to identify resilience subtypes that reflect the transactional dynamic in KRM.

A second limitation pertains to sampling bias. Our sample predominantly consists of white, female participants, which constrains the generalizability of the results. Recruiting participants through ResearchMatch.org may limit the available pool of potential subjects to those already enrolled in research projects funded by the NIMH, reducing the diversity of our prospective participant base. Additionally, relying solely on online self‐reported surveys for data collection might have inadvertently excluded individuals who experience technological disparities and limited the ecological validity of study results. For instance, the study measured perceived public stigma rather than objective public attitudes. Findings therefore reflect participants' subjective perceptions of stigma, which may be influenced by individual experiences and may not fully reflect societal attitudes.

Notably, participants' psychiatric diagnoses were self‐reported and not clinically verified. Although participants were permitted to endorse multiple diagnoses, and comorbidity was common (80.7%), participants relied on their own interpretation to identify a primary diagnosis when multiple diagnoses were selected, without being provided an explicit definition or standardized guidance. Consequently, participants may have differed in whether they selected a diagnosis based on symptom severity, functional impact, duration, or personal salience, which may have introduced diagnostic misclassification. Accordingly, findings that diverge from prior SMI literature (e.g., a profile that showed elevated stigma is also more likely to identify with anxiety disorders relative to bipolar disorder) should be interpreted cautiously, as they may reflect transdiagnostic or functional processes rather than diagnosis‐specific effects.

Moreover, the NIMH definition of SMI used to recruit participants for the current study is intentionally broad and anchored in functional impairment rather than specific diagnoses, resulting in a diagnostically heterogeneous sample with limited representation of individuals with schizophrenia spectrum disorders. This recruitment approach likely enhanced ecological validity, as real‐world services are often provided based on functional impairment and not assigned diagnosis, and may better capture transdiagnostic profiles of resilience in a population characterized by high psychiatric comorbidity (Halstead et al., 2024). However, this heterogeneity also limits the ability to examine diagnosis‐specific resilience processes, as study analyses did not differentiate between diagnostic groups when profiling. As a result, the obtained sample may not be representative of SMI groups defined by primary diagnosis or severe psychiatric symptoms. The study analyses did not differentiate between diagnostic groups while profiling. Presenting stressors (i.e., psychiatric symptom severity) and the environmental context (i.e., perceived public stigma) may vary meaningfully across diagnostic profiles and comorbidities, and these differences could not be fully disentangled in the current study. Accordingly, observed profile differences should be interpreted as cohort‐level averages among individuals with similar resilience patterns, with anticipated overlap between profiles. Future research should recruit from a more diagnostically diverse and representative sample and employ multi‐trait, multi‐method assessment to better differentiate diagnostic subgroups and determine whether similar resilience profiles emerge across specific SMI diagnoses.

A final limitation pertains to KRM itself, including the selection and operationalization of factors and the inherent ambiguity within some of Kumpfer's constructs (Kumpfer, 2002). KRM is a broad and integrative framework, and the variables selected for this study do not represent all subdomains described in KRM. For instance, internal resiliency factors in the original framework contain five subdomains (Kumpfer, 2002); however, only four measures were included in the current study. The physical resiliency subdomain was omitted due to limitations of self‐report measurement in capturing physical health and functioning. As a result, the operationalization of KRM in this study reflects a partial representation of the framework, which may limit the scope of the identified resilience profiles. Future researchers should carefully refer to the operational definitions used in the current study before adopting these results or making adaptations for their own use. Additional validation of KRM using alternative operationalizations of its constructs, including objective health indicators, is also recommended to examine model robustness and stability. Although KRM was selected for its relevance to SMI, alternative established resilience frameworks (Fergus & Zimmerman, 2005; Masten, 2018) may capture different aspects of adaptation, and comparative testing across models represents an important direction for future research.

## CONCLUSIONS

This study employed a latent profiling approach to examine all six components of KRM simultaneously among individuals with SMI, whereas previous studies only tested segments of the framework. Three distinct profiles (Maladaptive, Homeostatic, and Resilient) were identified that demonstrate the reciprocal relationships between internal resources and external challenges that influence resilience, with particular emphasis on the interactions between individuals and their environments. Additionally, the three profiles suggested indicators of presently high, moderate, and low resilience, revealing potential opportunities for effective interventions. The examination of demographic variables across the three profiles further identifies risk factors for diminished resilience. Building on these findings, researchers can further delineate the characteristics of these groups within individuals with SMI to design more targeted intervention plans to support their resilience and promote personal recovery.

## AUTHOR CONTRIBUTIONS

**Deyu Pan:** Conceptualization; investigation; writing – original draft; methodology; software; formal analysis; project administration. **Sang Qin:** Conceptualization; writing – original draft; writing – review and editing; validation. **Wilson J. Brown:** Conceptualization; writing – original draft; writing – review and editing. **Jessica Wojtalik:** Writing – original draft; writing – review and editing. **Charisse Nixon:** Writing – review and editing. **Beatrice Lee:** Writing – review and editing. **Emre Umucu:** Writing – review and editing.

## CONFLICT OF INTEREST STATEMENT

The authors declare that they have no known competing financial interests or personal relationships that could have appeared to influence the work reported in this paper.

## Data Availability

The data that support the findings of this study are available on request from the corresponding author. The data are not publicly available due to privacy or ethical restrictions.
